# Climate Change Projections for Stroke Incidence in Taiwan: Impact of 2 °C and 4 °C Global Warming Level

**DOI:** 10.1007/s44197-024-00289-3

**Published:** 2024-09-02

**Authors:** Wei-Te Wu, Miku Kono, Chuan-Pin Lee, Yu-Yin Chang, Yao-Hsu Yang, Ching-Chun Lin, Tzu-Ming Liu, Hsin-Chi Li, Yung-Ming Chen, Pau-Chung Chen

**Affiliations:** 1 National Institute of Environmental Health Sciences, National Health Research Institutes, No. 35, Keyan Rd., Zhunan Township, Miaoli County, 350401 Taiwan, ROC; 2 Institute of Environmental and Occupational Health Sciences, National Yang-Ming Chiao Tung University, Taipei, Taiwan; 3 Health Information and Epidemiology Laboratory, Chang Gung Memorial Hospital, Chiayi County, Taiwan; 4 Department of Traditional Chinese Medicine, Chang Gung Memorial Hospital, Chiayi County, Taiwan; 5 School of Traditional Chinese Medicine, College of Medicine, Chang Gung University, Taoyuan, Taiwan; 6 Institute of Environmental and Occupational Health Sciences, National Taiwan University College of Public Health, Taipei, Taiwan; 7 National Science and Technology Center for Disaster Reduction, New Taipei City, Taiwan; 8 Department of Public Health, National Taiwan University College of Public Health, Taipei, Taiwan; 9 Department of Environmental and Occupational Medicine, National Taiwan University Hospital, National Taiwan University College of Medicine, Taipei, Taiwan

**Keywords:** Climate change, Stroke, Case-crossover study, Precision environmental health

## Abstract

**Objectives:**

This study aimed to establish the exposure-lag-response effect between daily maximum temperature and stroke-related emergency department visits and to project heat-induced stroke impacts under global warming levels (GWL) of 2 °C and 4 °C.

**Methods:**

Stroke-related emergency department visits in Taiwan from 2001 to 2020 were identified using the National Health Insurance Research Database (NHIRD). The study population consisted of 1,100,074 initial stroke cases matched with 2,200,148 non-stroke controls. We employed Distributed Lag Nonlinear Models (DLNM) in a case-crossover study to investigate the association between temperature and stroke. Generalized Estimating Equations (GEE) models with a Poisson function were used to correlate high-temperature exposure with annual stroke incidence rates. Projections were made under two global warming scenarios, GWL 2.0 °C and 4.0 °C, using Coupled General Circulation Model (GCMs). Baseline data from 1995 to 2014 were transformed for spatial distribution at the township level. Geographic Information System (GIS) spatial analysis was performed using Quantum GIS 3.2.0 software.

**Results:**

DLNM exposure-lag-response effect revealed that daily maximum temperature exceeding 34 °C significantly increased the risk of stroke-related emergency department visits, particularly for ischemic stroke. Under the 2 °C GWL scenario, the frequency of days with temperatures surpassing 34 °C is projected to rise substantially by the median year of 2042, with a further increase to 92.6 ± 18.0 days/year by 2065 under the 4 °C GWL scenario. Ischemic stroke showed the highest increase in temperature-related incidence rates, notably rising from 7.80% under the GWL 2 °C to 36.06% under the GWL 4 °C. Specifically, the annual temperature-related incidence rate for ischemic stroke is expected to increase significantly by 2065. Regions such as Taichung, Hsinchu, Yilan, and Taitung demonstrated pronounced changes in heat-related ischemic stroke incidence under the GWL 4 °C.

**Conclusions:**

The findings emphasize the importance of addressing temperature-related stroke risks, particularly in regions projected to experience significant temperature increases. Effective mitigation strategies are crucial to reduce the impact of rising temperatures on stroke incidence and safeguard public health.

**Supplementary Information:**

The online version contains supplementary material available at 10.1007/s44197-024-00289-3.

## Introduction

Stroke ranks as the second-leading cause of death and the third-leading cause of disability-adjusted life years (DALYs) globally, according to the 2021 Global Burden of Disease Study [1]. Between 1990 and 2019, there was a substantial increase in stroke incidence, prevalence, deaths, and DALYs by 70.0%, 85.0%, 43.0%, and 32.0%, respectively [1]. Existing evidence highlights various physiological changes resulting from short-term ambient temperature variations, including alterations in blood pressure, inflammation, stress levels, and immune function [2–4]. Therefore, meteorological conditions play a significant role in the occurrence of cardiovascular and cerebrovascular diseases [5–9].

Over the past half-century, the global average temperature has steadily risen, with a recent acceleration due to global warming. Assessments by the Intergovernmental Panel on Climate Change (IPCC) indicate an increase in the global surface air temperature (GSAT) from 2001 to 2020 by 0.92 °C (0.68–1.17 °C) compared to the period from 1850 to 1900 [10]. Climate models have effectively reproduced climate averages from 1961 to 1990 and observed changes over time in GSAT. Projections for the period from 2081 to 2100 suggest higher GSAT averages under various emission scenarios, ranging from 0.2 °C to 1.0 °C in the low emission scenario SSP1-1.9 to 2.4 °C to 4.8 °C in the high emission scenario SSP5-8.5 [11]. In the SSP1-2.6, SSP2-4.5, and SSP3-7.0 scenarios, the corresponding ranges of likelihood are 0.5 °C to 1.5 °C, 1.2 °C to 2.6 °C, and 2.0 °C to 3.7 °C, respectively [11]. Recently, the Intergovernmental Panel on Climate Change Sixth 3 Assessment Report (AR6) uses 1.0 °C, 1.5 °C, 2.0 °C, 3.0 °C, and 4.0 °C as the primary global warming levels (GWLs). GWLs can distinguish global warming changes from regional climate responses, as scenario differences in response patterns at a given GWL are often smaller than model uncertainty and internal variability [12]. For many climate change indicators, such as seasonal and annual mean and extreme surface temperatures and precipitation, GWLs can accurately estimate changes independently of the emissions pathways or the timing of reaching the warming level [12]. Therefore, this study used GWLs of 2.0 °C and 4. 0 °C, reflecting increases in global surface temperature by 2 °C and 4 °C relative to the 1850–1900 period, to integrate climate information independently of specific scenarios.

Climate change has already led to shifts in cerebrovascular disease patterns, with this trend expected to persist and impact future disease burdens. However, most studies still rely on mortality as the primary outcome and utilize Representative Concentration Pathway (RCP) simulations, which may introduce bias due to disparities in healthcare access. To enhance our understanding of climate change’s effects on cerebrovascular diseases, we conducted a time-stratified case-crossover study to develop exposure-lag-response effect models linking ischemic and hemorrhagic stroke incidence with temperature. Additionally, we projected stroke incidence under GWLs of 2 °C and 4 °C and examined regions affected by long-term heat-induced stroke impacts.

## Methods

### Study Population and Design

The study utilized the National Health Insurance Research Database (NHIRD) to identify the study population in Taiwan. All patients (*n* = 1,781,712) with stroke-related emergency department visits, diagnosed with ICD-9 430 to 438 or ICD-10-CM code I60 to I69 for hemorrhagic stroke (430–432; I60-I62) and ischemic stroke (433–434; I63-I66) from January 1, 2001, to December 31, 2020, were included. Only initial stroke events were considered, leading to the exclusion of recurrent stroke events (*n* = 661,917). Patients residing on outlying islands (*n* = 5,321), with missing insurance data (*n* = 9,900), or who could not be matched with the two control days (*n* = 4,500) were also excluded. The final study population comprised 1,100,074 stroke-related emergency department visit cases, matched with 2,200,148 non-stroke dates as controls, from the NHIRD database. The study protocol was approved by the Research Ethics Committee of the National Health Research Institutes (Approval Number: EC1110702-E).

The study employed a case-crossover design to investigate the correlation between short-term meteorological parameters and stroke-related emergency department visits. In this design, each subject serves as their own control, with each stroke-related emergency department visit categorized as a case [13]. This methodological approach helps mitigate time-invariant factors such as gender and smoking habits [14], allowing for an assessment of the impact of extreme temperatures on subgroups based on characteristics such as age and health status [15, 16]. Two control days were matched to each case according to three and five weeks prior to the event of a stroke-related emergency department visit, considering the day of the week and season. This design minimizes potential biases that could arise after the events, such as disease recurrence. The hazard period of the case includes the event date (lag 0) and one to thirteen days prior to the event (lag 1 to lag 13), capturing the meteorological parameters such as daily mean temperature, daily maximum temperature, and daily minimum temperature. The control period comprises exactly three and five weeks preceding the event date.

### Environmental Exposure Modeling

Environmental exposure in this study focused on daily temperature as the primary variable. We utilized gridded daily observational data provided by the Taiwan Climate Change Projection Information and Adaptation Knowledge Platform (TCCIP) from 2001 to 2020, which including daily mean temperature, daily maximum temperature, and daily minimum temperature (Supplementary Table 1). The downscaling approach used for the TCCIP data involved integrating information from 953 meteorological stations across Taiwan, including the Central Weather Bureau, Agricultural Research and Extension Station, Taiwan Forestry Research Institute, Taiwan Agricultural Research Institute, Civil Aeronautics Administration, and Taiwan Power Company. This comprehensive data collection allowed for the creation of high-resolution temperature and rainfall grids at a 1 × 1 km spatial resolution, covering 120,701 grid points from 1960 to 2021, with a total of 22,646 time points across the entire Taiwan region (Supplementary Table 1) [17]. This method provides detailed local climate data but has several limitations. These include potential issues with extreme values, variations in temporal scale, and spatial discrepancies inherent in the original station data. To address these limitations, TCCIP conducted a cross-validation experiment to assess the uncertainties in generating gridded daily temperature data. In most of areas, the error was generally below 1 °C, whereas in parts of the Central Mountain Range, errors exceeded 4 °C, highlighting that lower station density leads to higher data uncertainty. However, since Taiwan’s major population centers are primarily located in flat areas, the data’s reliability remains robust in these regions.

To integrate these high-resolution climate data with regional-level data, we employed Geographic Information System (GIS) spatial statistic. This process involved averaging the data within each 0.01-degree grid across each township, allowing us to aggregate the gridded daily climate data to the 368-township level. As a result, we created a comprehensive daily township-level climate database spanning the period from 2001 to 2020, which was used for Generalized Estimating Equations (GEE) model.

Conditional logistic regression models were employed to quantify the association between stroke-related emergency department visits and daily temperature. To explore the possible nonlinear and lagged associations of temperature and stroke, we incorporated the distributed lag nonlinear model (DLNM) into the conditional logistic regression models [18]. This DLNM model creates a flexible cross-basis function for daily temperature and its changes, allowing for nonlinear exposure-response relationships in each lag day and nonlinear lagged effects [19]. In this study, we utilized a natural cubic *B*-spline with 4 degrees of freedom (df) in the exposure-response curve within the cross-basis function. Effects across different lag days were defined to exhibit linear trends, allowing for flexible lag effects at short delays. The main model in this study is represented by the following formula:

$$\:\text{l}\text{o}\text{g}\text{i}\text{t}\left[\text{P}\text{r}(Y=1)\right]={\sum\:}_{s}{\alpha\:}_{s}+f\left(T\right)$$

The function *f* is a bi-dimensional function describing the daily temperature *(T)* across lags. The parameter *α*_*s*_ is the intercept for strata. *Y* refers to the case or the control, and stratum is defined as the same days of the week in the same month and year for the same patient. A maximum lag of 13 days prior to stroke onset (lag 0–13 days) was utilized to thoroughly explore the lag pattern of short-term temperature exposure on stroke incidence, which was then applied in subsequent analyses.

Exposure-lag-response effect curves were plotted after adjusting for the distribution of temperature and its variations. To facilitate interpretation, the temperature associated with the lowest stroke risk in the exposure-response curve was selected as the reference, and findings were presented with odds ratios (ORs) and 95% confidence intervals (CIs) for stroke onset. Furthermore, stratified analyses were conducted by fitting separate models by age, season, and geographic area to identify susceptible populations.

### Daily Temperature and Stroke Incidence Projections

To determine risk temperature thresholds based on the exposure-lag-response effect curves mentioned previously, we identified temperature cutoff points where the impact on stroke incidence became significant. Specifically, we observed that daily maximum temperatures above 34 °C and daily minimum temperatures below 8 °C began to exhibit significant effects on stroke incidence. We utilized the coordinate reference system EPSG:3826 (TWD97 / 121 zone) and Quantum GIS 3.2.0 software for GIS spatial distribution analysis. Heatmaps of temperature variations, including maximum and minimum, were generated at the township level to illustrate the geographical distribution.

Subsequently, we utilized GEE with a Poisson function to establish models correlating the cumulative number of days exceeding the daily maximum temperature threshold with annual stroke incidence rates in each region. We incorporated autocorrelation between years using an AR covariance structure. The model with a 34 °C threshold is formulated as follows:

$$\eqalign{& {\rm{log}}\,{\rm{(}}{{\rm{n}}_{dise}})\sim\,offset\,{\rm{(log(}}{N_{pop}}))\, + \,{\beta _0}\, \cr & + \,{\beta _1}\,*\,{\rm{Da}}{{\rm{y}}_{ > 34}}\, + \,{\beta _{city}}\, + \,{\beta _{{\mathop{\rm int}} eraction}}\, \cr & *\,Da{y_{ > 34}} \cr} $$

- ${\beta _{{\mathop{\rm int}} eraction}}$ is the interaction effect of city and cumulative days of temperature higher than 34 °C in each year.
- Covariance structure: AR(1)

$$\left[ {\begin{array}{*{20}{c}}1&\rho & \cdots &{{\rho ^{years}}} \\ \rho &1& \ddots & \vdots \\ \vdots & \ddots & \ddots &\rho \\ {{\rho ^{years}}}& \ldots &\rho &1 \end{array}} \right]$$

Future temperature projections were derived from Coupled General Circulation Model (GCMs), which were downscaled and combined with five emission scenario pathways implemented in the Intergovernmental Panel on Climate Change Sixth Assessment Report (IPCC 6th). Data were collected from the Coupled Model Intercomparison Project Phase 6 (CMIP6) database, provided by the Earth System Grid Federation (ESGF, https://esgf-node.llnl.gov/projects/cmip6/) [20]. These Shared Socioeconomic Pathway (SSP) include extremely low emission (SSP1-1.9), low emission (SSP1-2.6), medium emission (SSP2-4.5), high emission (SSP3-7.0), and extremely high emission (SSP5-8.5). The datasets are well-documented and publicly accessible via TCCIP (https://tccip.ncdr.nat.gov.tw/ds_03_eng.aspx).

Theese GCMs were selected based on their inclusion in the CMIP6 dataset, ensuring a comprehensive representation of potential climate futures across various scenarios. Although a detailed performance comparison of the GCMs was not conducted for this specific study, the selected models are widely recognized and used in climate research, ensuring diverse and representative climate projections. Climate baseline data for the 1995–2014 period were used for spatial distribution transformation at the township level (ensemble size: 28 sets) (Supplementary Table 2). Future projections were made for spatial distribution changes at the township level under the scenarios of global warming by 2 °C (ensemble size: 86 sets) and 4 °C (ensemble size: 26 sets) (Supplementary Table 2) [10, 11].

All statistical analyses were performed in the R software (R package: dlnm, survival. version 3.4.4, R Foundation for Statistical Computing, Vienna, Austria) and SAS (SAS Institute Inc., Cary, North Carolina, U.S.A.). The statistical significance level was set at p-value < 0.05.

## Results

The study comprised 647,129 (58.8%) males and 452,945 (41.2%) females with emergency department visits for stroke, and most of the cases consisted of individuals above middle age, 344,265 (31.3%) in 45 to 64 years old and 538,944 (49.0%) in 65 to 84 years old groups. Hemorrhagic stroke accounted for 278,404 (25.3%) cases, while ischemic stroke accounted for 482,034 (43.8%) cases (Supplementary Table 3). Cases had higher mean, minimum, and maximum daily temperature parameters during spring and summer, while they exhibited lower values during autumn and winter compared to controls (Supplementary Table 4).

**Fig. 1:**
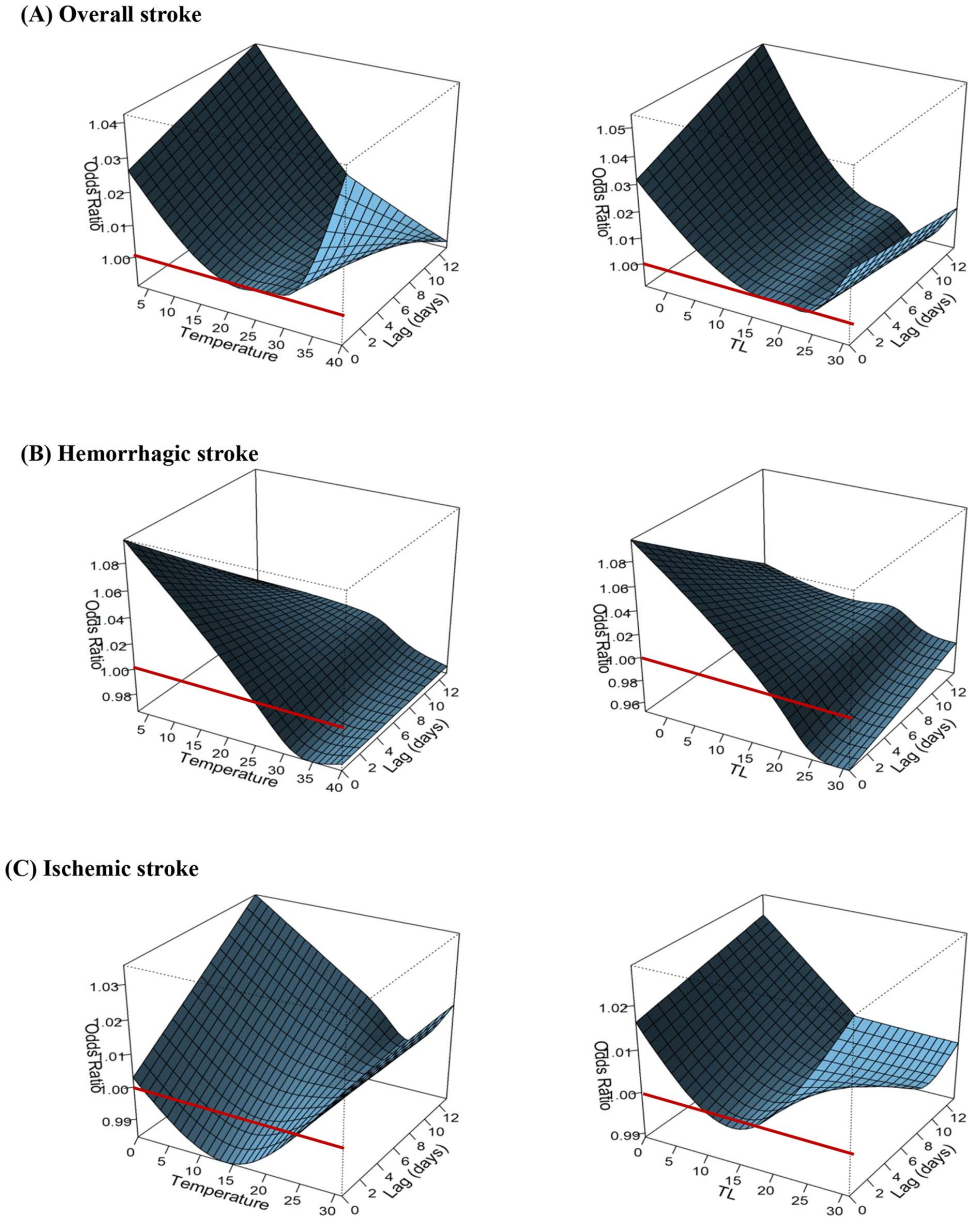
Exposure-response curves for daily maximum temperature (left) and daily minimum temperature (right) related incidence of stroke using the DLNM model across lag days 0–13: (**A**) overall stroke risk, (**B**) hemorrhagic stroke risk, (**C**) ischemic stroke risk (The red line represents OR = 1, and the black dashed line indicates the temperature at which statistical significance begins)

Figure 1 illustrates the DLNM exposure-lag-response effect of daily maximum and daily minimum temperatures on stroke-related emergency department visits. The risk of stroke-related emergency department visits showed a cut point at 34 °C, with a notable rise in risk observed when the temperature exceeded 35 °C (OR: 1.029, 95% CI: 1.007–1.051), reaching an OR of 1.250 (95% CI: 1.150–1.359) at 40 °C. Daily maximum temperature was associated with an increased risk of stroke incidence, with the delay effect observed from lag 1 to lag 8. Both heat and cold effects were evident in stroke occurrence, with acute ischemic stroke exhibiting the most pronounced heat effect. However, the incidence risk for hemorrhagic stroke increased with cold exposure, while the response for ischemic stroke was limited.

**Fig. 2:**
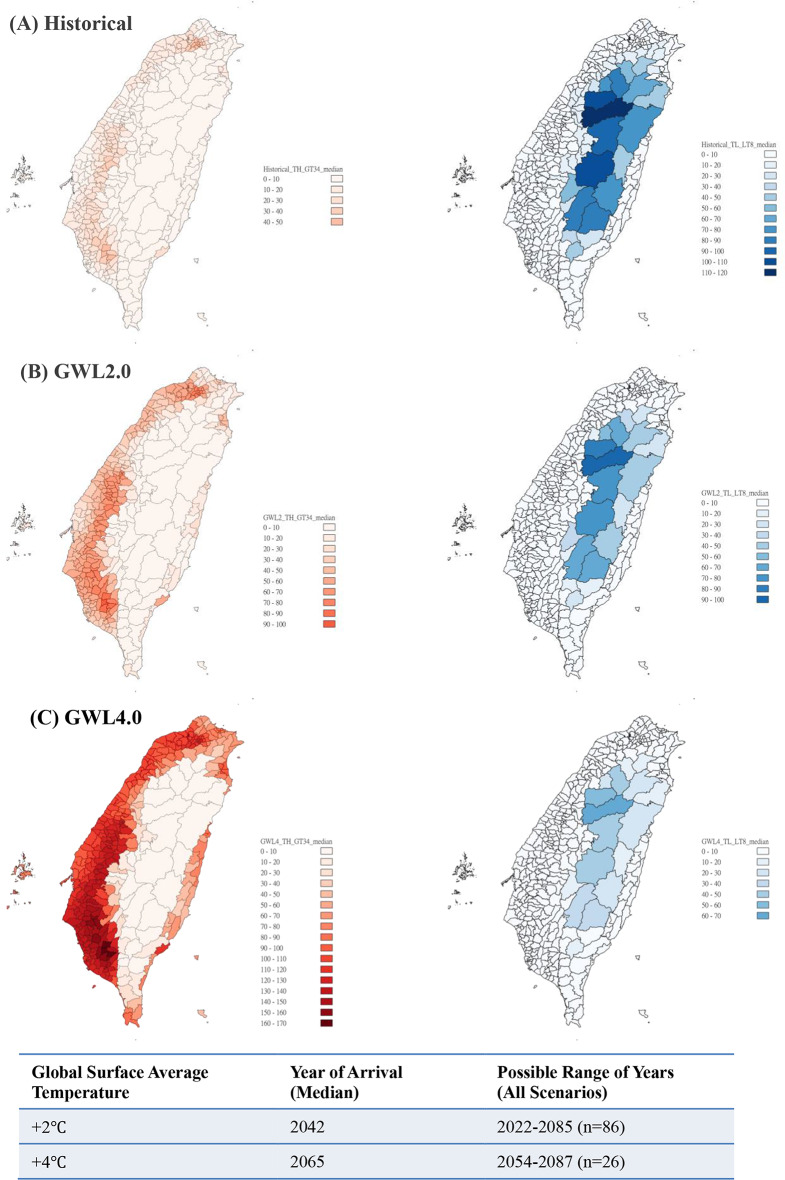
Distribution of days with daily maximum temperature > 34 °C (left) and daily minimum temperature < 8 °C (right) under the baseline period of AR6 (Historical) and at the global warming levels of 2 °C and 4 °C (GWL2.0 and GWL4.0)

Figure 2 presents projections based on temperature thresholds of 34 °C for daily maximum temperatures and 8 °C for daily minimum temperatures under the GWL 2 °C and GWL 4 °C scenarios. If global warming reaches 2 °C, the number of days with daily maximum temperatures exceeding 34 °C is expected to increase from 12.1 ± 1.9 days/year during the baseline period to 37.9 ± 8.6 days/year by the median year of 2042. With a 4 °C increase in global warming, this number is projected to rise to 92.6 ± 18.0 days/year by the median year of 2065. Conversely, the number of days with daily minimum temperatures below 8 °C is expected to continue decreasing under both GWL 2 °C and GWL 4 °C scenarios, shifting the focus primarily to the impact of high temperatures.

**Fig. 3:**
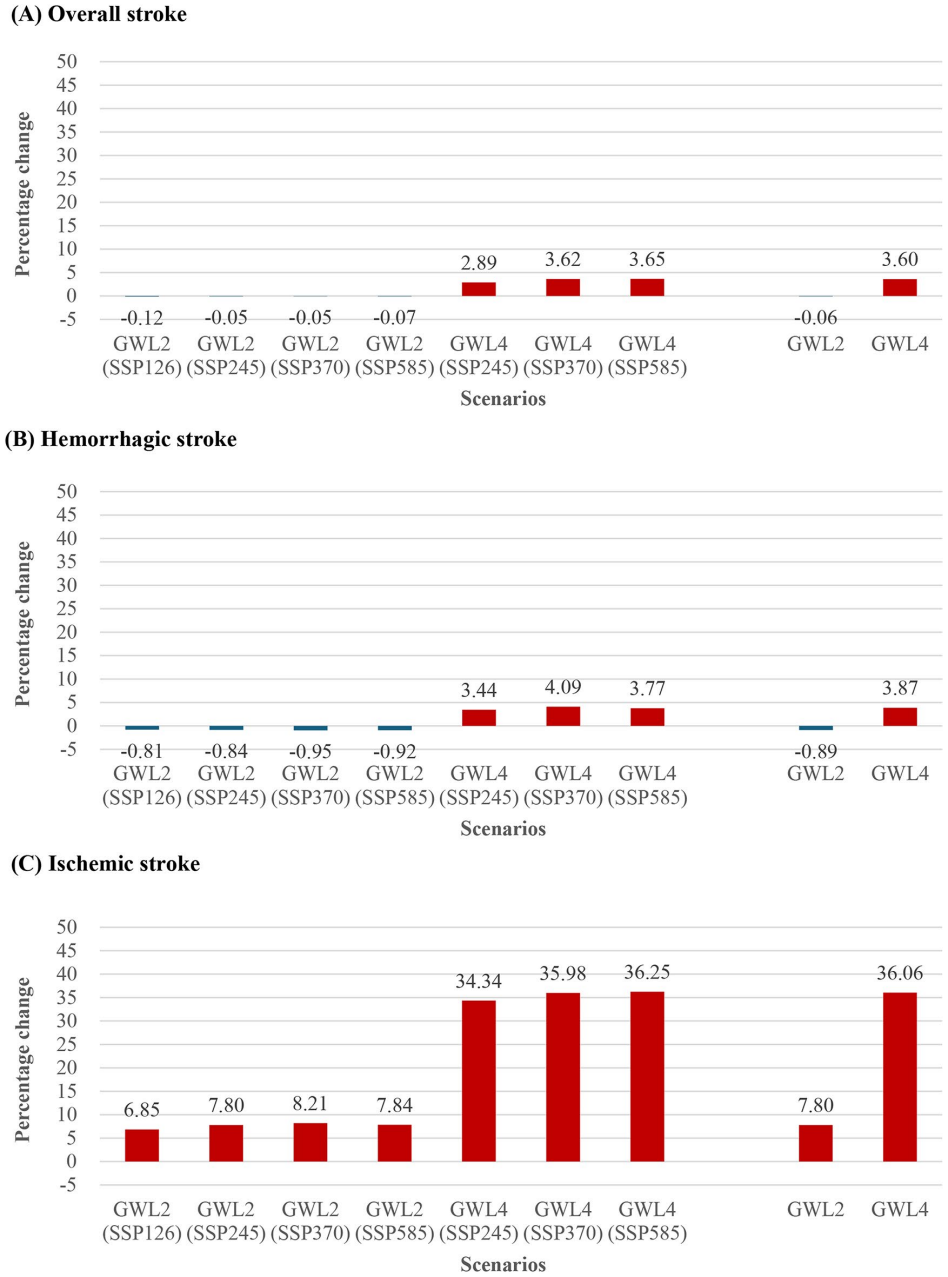
Percentage change in the daily maximum temperature-related incidence of stroke in the baseline (Historical) and future global warming levels of 2 °C and 4 °C scenarios (GWL2.0 and GWL4.0) with Shared Socioeconomic Pathway (SSP) using the Shared Socioeconomic Pathway (SSP) framework

Figure 3 illustrates the expected distribution of temperature-related incidence rates for ischemic and hemorrhagic stroke under the GWL 2 °C and GWL 4 °C scenarios. The overall net incidence rates of stroke related to temperatures decreased slightly by 0.06% and 0.89% for ischemic and hemorrhagic stroke, respectively, under the GWL 2 °C scenario. However, under the GWL 4 °C scenario, these rates increased by 3.60% and 3.87%, respectively. Among the temperature-related net incidence rates, ischemic stroke showed the highest increase, rising from 7.80% under the GWL 2 °C to 36.06% under the GWL 4 °C. Specifically, for ischemic stroke under the GWL 2 °C, the annual temperature-related incidence rate increased from 102.02 ± 0.48 per 100,000 person-years in the baseline period (1995–2014) to 119.96 ± 3.16 per 100,000 person-years by the median year of 2042. Under the GWL 4 °C, this rate increased further to 138.29 ± 8.78 per 100,000 person-years by the median year of 2065.

**Fig. 4:**
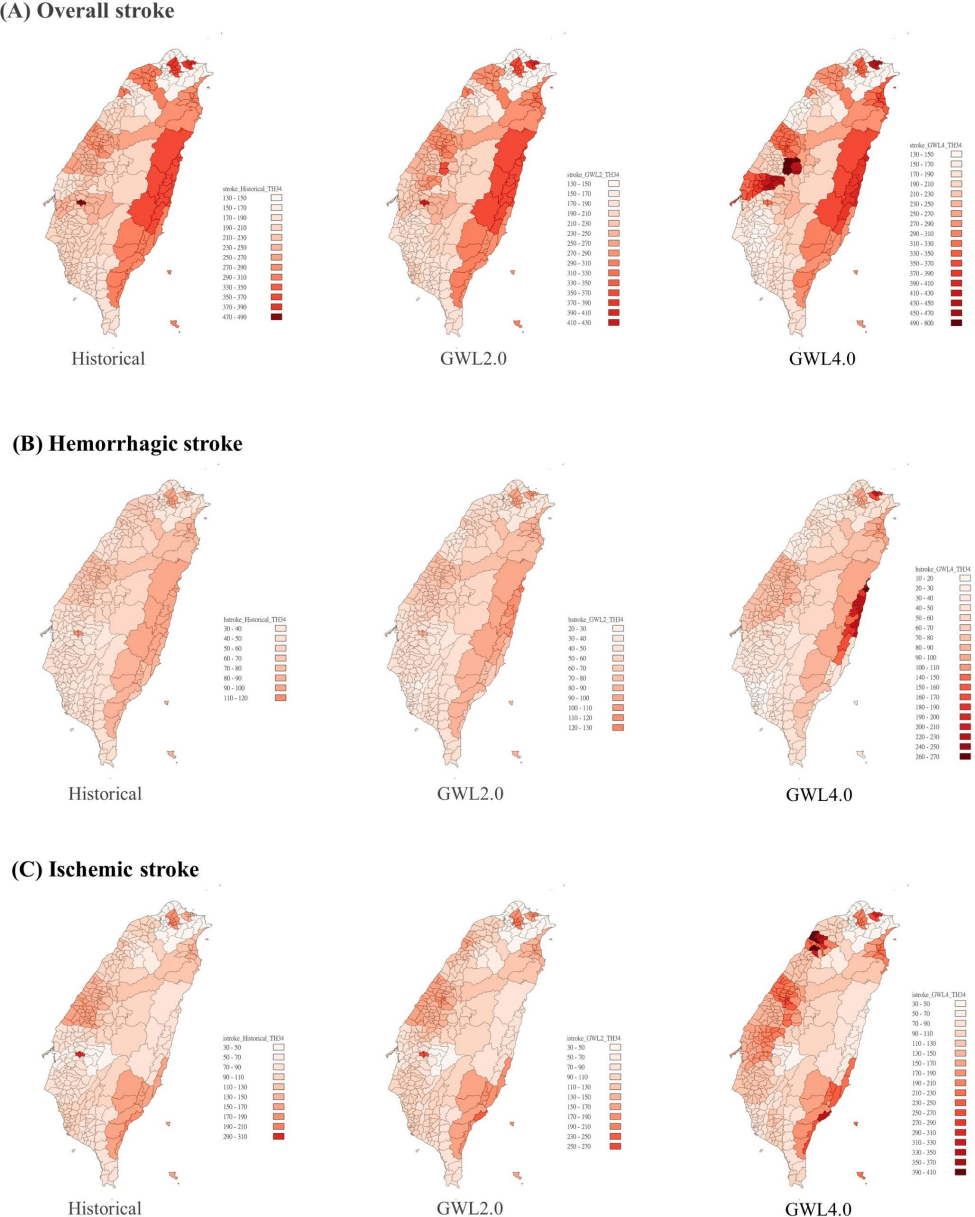
Distribution of changes in stroke incidence estimated by the GEE model with the baseline (Historical) and future global warming levels of 2°C and 4°C (GWL2.0 and GWL4.0), using the daily maximum temperature threshold of 34°C

Using the GEE model with the 34 °C daily maximum temperature threshold, projections indicated the most significant changes in heat-related stroke incidence in Nantou and Mingjian in the central area, followed by Cidong, Dounan, Douliu, and Linnei in the central-south area under the GWL 2 °C scenario. With the GWL 4 °C increase in global warming, the impact of these regions is expected to expand further. Additionally, under the GWL 4 °C scenario, the most noticeable changes in heat-related ischemic stroke incidence were observed in the Taichung, Hsinchu, Yilan, and Taitung regions (Fig. 4).

## Discussion

This study represents the first endeavor to establish the exposure-lag-response effect relationship between stroke incidence and temperature, utilizing two GWL scenarios, 2.0 °C and 4.0 °C, with 31 GCMs and four SSP scenarios to predict variations in heat-related stroke incidence. We found that ischemic stroke exhibited the highest increase in temperature-related incidence rates, notably rising from 7.80% under the GWL 2 °C to 36.06% under the GWL 4 °C, with a significant annual temperature-related incidence rate increase projected by 2065. Regions such as Taichung, Hsinchu, Yilan, and Taitung demonstrated pronounced changes in heat-related ischemic stroke incidence under the GWL 4 °C. Climate change has already led to shifts in ischemic stroke incidence, underscoring the need for public health interventions informed by estimated climate change impacts.

### Climate Change Projections for Stroke Incidence

Our study revealed that ischemic stroke demonstrated the most significant increase in temperature-related incidence rates. Notably, the GWL 4 °C SSP585 scenario presented the highest burden, reflecting an unchecked trajectory characterized by elevated greenhouse gas emissions, population growth, fossil fuel consumption, and air pollution [11]. These findings underscore the urgent need for targeted mitigation strategies to combat global warming and mitigate adverse health effects. For instance, our analysis suggests that implementing mitigation measures could yield substantial health benefits, with ischemic stroke incidence rates projected to decrease by 28.04–29.4% compared to various GWL 2 °C SSP scenarios.

Consistent with previous research, it suggests that rising temperatures may contribute to increased mortality rates from cerebrovascular diseases [21], particularly ischemic stroke. However, previous studies have primarily focused on mortality rates rather than stroke incidence and have utilized RCP simulations with aging being a significant consideration. Under the RCP8.5 scenario, it is anticipated that cardiovascular disease mortality rates in Beijing will rise by 16.6%, 73.8%, and 134.0% in 2020, 2050, and 2080, respectively [22]. Another study conducted in Brazil used different climate change scenarios and regional climate models to predict the impact of climate change on cardiovascular mortality rates in 21 cities from 2010 to 2099. According to the RCP8.5 scenario, compared to the period from 2010 to 2019, the temperature-related mortality fractions in 2090–2099 are expected to increase by 8.6% and 1.7% under Eta-HadGEM2-ES and Eta-MIROC5, respectively. Under the RCP4.5 scenario, these values are projected to be 0.7% and − 0.6%, respectively [23].

For ischemic stroke, the annual temperature-related incidence rate increased from 102.02 ± 0.48 per 100,000 person-years during the baseline period (1995–2014) to 119.96 ± 3.16 per 100,000 person-years by the median year of 2042 under the GWL 2 °C. Under the GWL 4 °C, this rate further increased to 138.29 ± 8.78 per 100,000 person-years by the median year of 2065. In comparison to other studies, a study conducted in China, covering 161 districts and counties, projected heat-related attributable deaths from cardiovascular disease in the 2090s under RCP8.5, which were notably higher than those from respiratory disease, totaling 122,006 (95% eCI: 45,041–197,769) and 47,168 (95% eCI: −9968-89,509) deaths, respectively [24]. Additionally, another study in China projected environmental temperatures from 2016 to 2070 under the RCP4.5 and RCP8.5 scenarios. Compared to the baseline period of 1981–2005, the average annual mortality rate for cardiovascular diseases from May to September in Jiangsu Province increased. Under the RCP4.5 scenario, there was an increase of 3.3 deaths per 100,000 people from 2016 to 2040 and 6.8 deaths per 100,000 people from 2041 to 2065. Under the RCP8.5 scenario, there was an increase of 2.2 deaths per 100,000 people from 2016 to 2040 and 11.4 deaths per 100,000 people from 2041 to 2065 [25].

Our projections revealed the most notable shifts in heat-related ischemic stroke incidence in the Taichung, Hsinchu, Yilan, and Taitung regions under the GWL 4 °C. Our study identified significant spatial variations in future temperature changes, particularly under the GWL 4 °C. This divergence may stem from heightened heat susceptibility among local populations and relatively rapid temperature escalations in these areas. However, the interplays of swift urbanization and the urban heat island effect could expedite temperature spikes, potentially heightening the health hazards of future heat exposure in these highly developed regions like Taichung and Hsinchu. These insights inform the development of targeted local intervention strategies aimed at mitigating the future stroke burden linked to heat.

### Temperature-Lag-Stroke Effect

Our findings highlight a clear relationship between ambient temperature and stroke risk over several consecutive days. The DLNM exposure-lag-response effect revealed an elevated risk of stroke incidence starting from the threshold of 34 °C, with effects persisting from lag 1 to lag 8. Consistent with previous research on cardiovascular disease mortality, we observed a U-shaped response curve, indicating increased risk both above and below the optimal temperature [26]. Exposure to high temperatures can trigger vasodilation, increased peripheral circulation, and sweating, leading to dehydration, increased blood viscosity, and elevated cholesterol levels [27, 28]. However, induced hypercoagulability due to hemoconcentration and hyperviscosity may increase the risk of ischemic stroke [27, 29]. Conversely, exposure to low temperatures or temperature decreases may elevate blood pressure, a response mediated by sympathetic nervous system activation [2, 30], potentially exacerbating aortic wall damage and increasing the risk of hemorrhagic stroke [31]. We observed a relatively larger heat effect for ischemic stroke and a cold effect for hemorrhagic stroke, consistent with previous research [32]. Despite Taiwan’s implementation of heat alert levels in response to climate change health impacts, there exists a disparity between the recommended daily maximum thresholds and the 34 °C threshold identified in our study as indicative of stroke onset.

### Adaptation Strategies

The IPCC outlines climate change adaptation strategies to mitigate human health risks, including cardiovascular diseases, in its Sixth Assessment Report Working Group II. These strategies encompass improving health infrastructure availability, enhancing access to healthcare, and implementing disaster warnings [33]. Successful adaptation measures can reduce climate-related health risks. For instance, urban green spaces can alleviate the urban heat island effect and improve mental and physical health [34]. Strengthening health infrastructure and access to healthcare can bolster resilience among vulnerable groups [35]. Local cooling and warning systems can minimize mortality, morbidity, and potential infectious disease outbreaks [34, 35]. Therefore, proactive measures are recommended to prevent climate change’s impact on cardiovascular health. These measures include early adoption of evidence-based lifestyle changes, medication adjustments, cardiovascular care, and communication recommendations to aid clinicians and healthcare professionals in preventing climate-related heart diseases. At national and institutional levels, hot weather health action plans should be implemented, offering access to cool environments, behavioral advice, medication adjustments, and effective treatment for cardiovascular risk patients. Physicians should identify high-risk patients, provide behavioral advice, and adjust medication treatments based on high temperatures and heatwaves. Cardiologists are encouraged to provide specific consultations to patients regarding climate change’s health impacts, conduct pre-summer medical assessments, and offer advice on coping with high temperatures to enhance awareness and protective measures. Finally, individuals are advised to adopt proactive behaviors during hot weather, such as limiting outdoor activities during peak heat, utilizing air conditioning and ventilation, wearing loose clothing and hats, and staying hydrated by drinking ample water.

Strengths and Limitations.

This study boasts several strengths. It marks the first attempt to establish the exposure-lag-response effect relationship between stroke incidence and temperature, utilizing two GWLs, 2.0 °C and 4.0 °C, with 31 GCMs and four SSP scenarios to predict variations in heat-related stroke incidence. Unlike the previous RCP simulations, which solely represented different emission concentration levels, SSP assessments incorporate factors such as population, education, urbanization level, and GDP, offering a more comprehensive evaluation. The use of a time-stratified case-crossover design and DLNM analysis in conditional logistic regression enabled the capture of exposure-lag-response effects, enhancing our understanding of the relationship between ambient temperature and stroke risk. Furthermore, the inclusion of a large study population in Taiwan over a decade minimized random error and allowed for stratified analysis by season and geographic regions. Additionally, exploring the temperature effect on different stroke types, hemorrhagic and ischemic, shed light on the varying temperature fluctuation effects on stroke incidences. Finally, we utilized a multi-model ensemble approach to reduce biases and uncertainties across different models while optimizing computational resources. However, this approach may underestimate the impact of climate change, as it might overlook high-frequency information [36, 37].

However, there are some limitations to be acknowledged in the present study. Firstly, exposure measurement errors are inevitable due to individuals’ movements and daily activities, potentially introducing inaccuracies in temperature exposure assessment, albeit these errors may be randomly distributed. Additionally, there is a possibility of omitting some confounding variables in the analysis. While these confounding factors may vary within each time stratum, it is assumed that their significant changes within a single time stratum are unlikely. Thirdly, the scenarios may not fully represent the proportion of elderly individuals and population size in specific regions, potentially resulting in conservative estimates of future stroke incidence related to temperature. Further research is needed to accurately quantify future population changes. In Taiwan, demographic shifts are underway, with the elderly population projected to exceed 20% by 2039 and reach 43.6% by 2070, marking a transition to a super-aged society [38]. Unfortunately, Taiwan lacks data for the end of this century, hindering comparisons with alternative population scenarios. While future population aging may lead to higher projected values, our study can only provide quantitative evidence for stroke predictions under scenarios with and without population changes. Fourthly, the human ability to adapt to warmer climates may be enhanced [39], potentially leading to an overestimation of heat-related disease occurrences in our study. We assume that the shape of the exposure-response curve for temperature-mortality associations and climate adaptation capabilities will remain consistent with levels observed in the 2001–2010 period. Therefore, our estimates should be interpreted as potential impacts of future high temperatures under hypothetical scenarios, rather than predictions of excessively high incidences in the future [40]. Final, we employed a multi-model ensemble approach to minimize biases and uncertainties across different models while also optimizing computational resources. However, it’s important to note that this method may underestimate certain impacts of climate change, particularly those related to high-frequency variations [36, 37].

## Conclusion

Climate change has shifted ischemic heart disease and stroke incidence rates, emphasizing the necessity for public health interventions informed by projected climate change impacts. Ischemic stroke rates notably rose from 7.80% under the GWL 2 °C to 36.06% under the GWL 4 °C, with projected annual temperature-related incidence rate increases by 2065. Our findings advocate for initiatives aimed at reducing stroke occurrence, especially in regions expected to face significant temperature increases.

## Electronic Supplementary Material

Below is the link to the electronic supplementary material.

Supplementary Material 1

## Data Availability

No datasets were generated or analysed during the current study.
