# Supplementary material for: Climate Change Projections for Stroke Incidence in Taiwan: Impact of 2 °C and 4 °C Global Warming Level
Source: J Epidemiol Glob Health. 2024 Sep 2;14(3):1319–31. doi: 10.1007/s44197-024-00289-3 (PMC11442790; doi:10.1007/s44197-024-00289-3)
Supplement: Supplementary file 1 — Supplementary Material 1 [file 44197_2024_289_MOESM1_ESM.docx]

Supplementary Table 1. List of TCCIP's gridded observed temperatures

| Time-resolution | Space-resolution | Time period | Meteorological parameters | Source |
| --- | --- | --- | --- | --- |
| Daily | 0.01ﾟapproximately 1 km²) | 1960-2021 | Daily mean temperature, daily maximum temperature, and daily minimum temperature | https://tccip.ncdr.nat.gov.tw/ds_03_eng.aspx |

Data Generation Description (Chinese document): https://tccip.ncdr.nat.gov.tw/upload/data_profile/20220706114121.pdf https://tccip.ncdr.nat.gov.tw/upload/data_document/20220708162241.pdf

Supplementary Table 2. List of CMIP6 models which were adopted in calculating future projections

(A) 2°C global warming scenario

| **CMIP 6 model** | **Research Center (Country)** | **Space-resolution (lon×lat)** | **historical** | **SSP126** | **SSP245** | **SSP370** | **SSP585** |
| --- | --- | --- | --- | --- | --- | --- | --- |
| ACCESS-CM2 | CSIRO-ARCCSS (Australia) | 192×144 | 1995-2014 | 2033-2052 | 2031-2050 | 2030-2049 | 2029-2048 |
| ACCESS-ESM1-5 | CSIRO (Australia) | 192×145 | 1995-2014 | 2064-2083 | 2036-2055 | 2039-2058 | 2030-2049 |
| AWI-CM-1-1-MR | AWI (Germany) | 384×192 | 1995-2014 | 2041-2060 | 2030-2049 | 2028-2047 | 2027-2046 |
| BCC-CSM2-MR | BCC (China) | 320×160 | 1995-2014 |  | 2048-2067 | 2037-2056 | 2034-2053 |
| CanESM5 | CCCma (Canada) | 128×64 | 1995-2014 | 2017-2036 | 2015-2034 | 2014-2033 | 2013-2032 |
| CMCC-ESM2 | CMCC (Italy) | 288×192 | 1995-2014 | 2033-2052 | 2031-2050 | 2032-2051 | 2030-2049 |
| EC-Earth3 | EC-Earth-Consortium(Europe) | 512×256 | 1995-2014 | 2034-2053 | 2035-2054 | 2029-2048 | 2026-2045 |
| EC-Earth3-AerChem | EC-Earth-Consortium(Europe) | 512×256 | 1995-2014 |  |  | 2038-2057 |  |
| EC-Earth3-CC | EC-Earth-Consortium(Europe) | 512×256 | 1995-2014 |  | 2026-2045 |  | 2027-2046 |
| EC-Earth3-Veg | EC-Earth-Consortium(Europe) | 512×256 | 1995-2014 | 2020-2039 | 2024-2043 | 2023-2042 | 2018-2037 |
| EC-Earth3-Veg-LR | EC-Earth-Consortium(Europe) | 320×160 | 1995-2014 |  | 2040-2059 | 2036-2055 | 2032-2051 |
| FGOALS-g3 | CAS (China) | 180×80 | 1995-2014 |  | 2054-2073 | 2036-2055 | 2037-2056 |
| GFDL-CM4 | NOAA-GFDL (USA) | 288×180 | 1995-2014 |  | 2040-2059 |  | 2032-2051 |
| GFDL-ESM4 | NOAA-GFDL (USA) | 288×180 | 1995-2014 |  | 2064-2083 | 2048-2067 | 2043-2062 |
| IITM-ESM | CCCR-IITM (India) | 192×94 | 1995-2014 |  |  |  |  |
| INM-CM4-8 | INM (Russia) | 180×120 | 1995-2014 |  | 2054-2073 | 2043-2062 | 2037-2056 |
| INM-CM5-0 | INM (Russia) | 180×120 | 1995-2014 |  | 2063-2082 | 2041-2060 | 2037-2056 |
| IPSL-CM6A-LR | IPSL (France) | 144×143 | 1995-2014 | 2029-2048 | 2024-2043 | 2025-2044 | 2025-2044 |
| KACE-1-0-G | NIMS-KMA (Korea) | 192×144 | 1995-2014 | 2015-2034 | 2014-2033 | 2015-2034 | 2014-2033 |
| KIOST-ESM | KIOST (Korea) | 192×96 | 1995-2014 |  | 2031-2050 |  | 2029-2048 |
| MIROC6 | MIROC (Japan) | 256×128 | 1995-2014 |  | 2064-2083 | 2050-2069 | 2044-2063 |
| MPI-ESM1-2-HR | DKRZ (Germany) | 384×192 | 1995-2014 |  | 2054-2073 | 2041-2060 | 2040-2059 |
| MPI-ESM1-2-LR | MPI-M (Germany) | 192×96 | 1995-2014 |  | 2048-2067 | 2043-2062 | 2039-2058 |
| MRI-ESM2-0 | MRI (Japan) | 320×160 | 1995-2014 |  | 2040-2059 | 2036-2055 | 2029-2048 |
| NESM3 | NUIST (China) | 192×96 | 1995-2014 | 2040-2059 | 2034-2053 |  | 2025-2044 |
| NorESM2-LM | NCC (Norway) | 144×96 | 1995-2014 |  | 2076-2095 | 2060-2079 | 2047-2066 |
| NorESM2-MM | NCC (Norway) | 288×192 | 1995-2014 |  | 2069-2088 | 2053-2072 | 2045-2064 |
| TaiESM1 | AS-RCEC (Taiwan) | 288×192 | 1995-2014 | 2031-2050 | 2034-2053 | 2034-2053 | 2027-2046 |
| **Total** |  |  | **28** | **11** | **26** | **23** | **26** |

(B) 4°C global warming scenario

| **CMIP 6 model** | **Research Center (Country)** | **Space-resolution (lon×lat)** | **SSP245** | **SSP370** | **SSP585** |
| --- | --- | --- | --- | --- | --- |
| ACCESS-CM2 | CSIRO-ARCCSS (Australia) | 192×144 |  | 2073-2092 | 2062-2081 |
| ACCESS-ESM1-5 | CSIRO (Australia) | 192×145 |  |  | 2069-2088 |
| AWI-CM-1-1-MR | AWI (Germany) | 384×192 |  |  | 2070-2089 |
| CanESM5 | CCCma (Canada) | 128×64 | 2074-2093 | 2050-2069 | 2045-2064 |
| CMCC-ESM2 | CMCC (Italy) | 288×192 |  | 2078-2097 | 2061-2080 |
| EC-Earth3 | EC-Earth-Consortium(Europe) | 512×256 |  | 2075-2094 | 2064-2083 |
| EC-Earth3-AerChem | EC-Earth-Consortium(Europe) | 512×256 |  | 2074-2093 |  |
| EC-Earth3-CC | EC-Earth-Consortium(Europe) | 512×256 |  |  | 2062-2081 |
| EC-Earth3-Veg | EC-Earth-Consortium(Europe) | 512×256 |  | 2067-2086 | 2058-2077 |
| EC-Earth3-Veg-LR | EC-Earth-Consortium(Europe) | 320×160 |  | 2078-2097 | 2066-2085 |
| GFDL-CM4 | NOAA-GFDL (USA) | 288×180 |  |  | 2070-2089 |
| IPSL-CM6A-LR | IPSL (France) | 144×143 |  | 2067-2086 | 2057-2076 |
| KACE-1-0-G | NIMS-KMA (Korea) | 192×144 |  | 2063-2082 | 2053-2072 |
| MPI-ESM1-2-0 | MRI (Japan) | 320×160 |  |  | 2074-2093 |
| NESM3 | NUIST (China) | 192×96 |  |  | 2063-2083 |
| TaiESM1 | AS-RCEC (Taiwan) | 288×192 |  | 2072-2091 | 2058-2077 |
| **Total** |  |  | **1** | **10** | **15** |

Meteorological parameters: Daily mean temperature, daily maximum temperature, and daily minimum temperature

Source: https://tccip.ncdr.nat.gov.tw/ds_03_eng.aspx

Data Generation Description (Chinese document): https://tccip.ncdr.nat.gov.tw/upload/data_profile/20221228143303.pdf

Supplementary Table 3. Demographic characteristics of cases with emergency department visits for stroke in Taiwan from 2001-2020

|  | Cases with emergency department visits for stroke  (n =1100074) | |
| --- | --- | --- |
|  | *n* | *%* |
| Gender |  |  |
| Male | 647129 | 58.8% |
| Female | 452945 | 41.2% |
| Age (year) |  |  |
| 0-5 | 2386 | 0.2% |
| 6-17 | 6064 | 0.6% |
| 8-44 | 94483 | 8.6% |
| 45-64 | 344265 | 31.3% |
| 65-84 | 538944 | 49.0% |
| ≥ 85 | 113932 | 10.4% |
| Seasons |  |  |
| Spring (Mar-May) | 278625 | 25.3% |
| Summer (Jun-Aug) | 270045 | 24.5% |
| Autumn (Sep-Nov) | 268371 | 24.4% |
| Winter (Dec-Feb) | 283033 | 25.7% |
| Study areas |  |  |
| Central | 280937 | 25.5% |
| North | 510933 | 46.4% |
| East | 37918 | 3.4% |
| South | 270286 | 24.6% |
| Types of strokes |  |  |
| Hemorrhagic stroke | 278404 | 25.3% |
| Ischemic stroke | 482034 | 43.8% |
| missing | 339636 | 30.9% |

Supplementary Table 4. Distribution of daily temperature parameters in cases with emergency department visits for stroke and their controls

|  | Cases with emergency department visits for stroke | | | | | | | | | Controls | | | | | | | | |
| --- | --- | --- | --- | --- | --- | --- | --- | --- | --- | --- | --- | --- | --- | --- | --- | --- | --- | --- |
|  | *N* | *Mean* | *SD* | *Min* | *Q1* | *Q2* | *Q3* | *Max* | *IQR* | *N* | *Mean* | *SD* | *Min* | *Q1* | *Q2* | *Q3* | *Max* | *IQR* |
| Daily min temperature (°C) |  |  |  |  |  |  |  |  |  |  |  |  |  |  |  |  |  |  |
| Overall | 1100074 | 20.1 | 5.1 | -3.1 | 16.1 | 20.8 | 24.5 | 30.8 | 8.5 | 2200148 | 20.1 | 5.1 | -1.2 | 16.1 | 20.8 | 24.5 | 30.8 | 8.4 |
| Seasons |  |  |  |  |  |  |  |  |  |  |  |  |  |  |  |  |  |  |
| Spring (Mar-May) | 278625 | 19.3 | 4.0 | 1.2 | 16.5 | 19.7 | 22.4 | 28.9 | 6.0 | 557250 | 17.0 | 3.9 | -0.2 | 14.3 | 17.0 | 19.9 | 27.9 | 5.6 |
| Summer (Jun-Aug) | 270045 | 25.3 | 1.6 | 13.3 | 24.4 | 25.4 | 26.4 | 30.8 | 2.0 | 540090 | 24.6 | 2.1 | 11.6 | 23.5 | 24.9 | 26.1 | 30.8 | 2.6 |
| Autumn (Sep-Nov) | 268371 | 21.7 | 3.2 | 6.0 | 19.6 | 22.0 | 24.2 | 29.6 | 4.6 | 536742 | 23.5 | 2.6 | 9.0 | 21.9 | 24.0 | 25.4 | 30.6 | 3.5 |
| Winter (Dec-Feb) | 283033 | 14.2 | 3.1 | -3.1 | 12.2 | 14.4 | 16.4 | 23.6 | 4.2 | 566066 | 15.4 | 3.7 | -1.2 | 12.9 | 15.5 | 18.0 | 26.4 | 5.1 |
| Daily max temperature (°C) |  |  |  |  |  |  |  |  |  |  |  |  |  |  |  |  |  |  |
| Overall | 1100074 | 26.9 | 5.6 | 3.1 | 23.1 | 27.9 | 31.6 | 39.0 | 8.5 | 2200148 | 26.9 | 5.6 | 3.1 | 23.0 | 27.8 | 31.6 | 39.0 | 8.5 |
| Seasons |  |  |  |  |  |  |  |  |  |  |  |  |  |  |  |  |  |  |
| Spring (Mar-May) | 278625 | 26.4 | 4.7 | 6.9 | 23.6 | 27.2 | 30.0 | 39.0 | 6.4 | 557250 | 24.2 | 5.0 | 5.6 | 20.8 | 24.9 | 28.0 | 39.0 | 7.2 |
| Summer (Jun-Aug) | 270045 | 32.2 | 2.1 | 17.1 | 31.3 | 32.6 | 33.6 | 38.7 | 2.3 | 540090 | 31.5 | 2.7 | 16.4 | 30.2 | 32.1 | 33.3 | 38.7 | 3.1 |
| Autumn (Sep-Nov) | 268371 | 28.2 | 3.8 | 12.4 | 25.8 | 28.8 | 31.1 | 37.8 | 5.3 | 536742 | 30.1 | 3.2 | 14.7 | 28.2 | 30.8 | 32.4 | 38.5 | 4.2 |
| Winter (Dec-Feb) | 283033 | 21.1 | 4.4 | 3.1 | 18.1 | 21.4 | 24.4 | 34.2 | 6.3 | 566066 | 22.0 | 4.4 | 3.1 | 19.0 | 22.3 | 25.3 | 33.8 | 6.3 |

Min: minimum; Max: maximum; IQR: interquartile range
